# Perioperative Intensive Smoking Cessation Intervention Among Smokers Who Underwent Transurethral Resection of Bladder Tumor (TURBT) in Two Different Settings: A Randomized Controlled Trial

**DOI:** 10.3390/cancers17040713

**Published:** 2025-02-19

**Authors:** Line N. Lydom, Susanne V. Lauridsen, Mie S. Liljendahl, Anne V. Schmedes, Ulla N. Joensen, Hanne Tønnesen

**Affiliations:** 1 WHO-CC/Clinical Health Promotion Centre, The Parker Institute, Bispebjerg and Frederiksberg Hospital, University of Copenhagen, Nordre Fasanvej 57-59, Vej 8, Indgang 19, DK-2000 Frederiksberg, Denmark; 2 Department of Surgery and Urology, Copenhagen University Hospital—Herlev and Gentofte, Borgmester Ib Juuls Vej 1, DK-2730 Herlev, Denmark; 3 Department of Clinical Medicine, University of Copenhagen, Blegdamsvej 3B, DK-2200 Copenhagen N, Denmark; 4 Department of Biochemistry and Immunology, Lillebaelt Hospital, University Hospital of Southern Denmark, Beriderbakken 4, DK-7100 Vejle, Denmark; anne.vibeke.schmedes@rsyd.dk; 5 Department of Urology, Rigshospitalet, University of Copenhagen, Inge Lehmanns Vej 7, DK-2100 Copenhagen Ø, Denmark

**Keywords:** smoking, smoking cessation, non-muscle invasive bladder neoplasms, GSP

## Abstract

**Background/Objective:** Smoking is an individual risk factor for bladder cancer. Many patients are still smoking at the time of their diagnosis, yet few trials have examined smoking cessation interventions (SCIs) in relation to transurethral resection of the bladder tumor (TURBT). This study therefore aimed to compare the efficacy of a hospital-based 6-week intensive SCI with standard treatment among this patient group. **Methods:** A parallel-arm randomized controlled trial was conducted from December 2021 to March 2024 at two university hospital urology departments in Denmark. A total of 38 daily smokers undergoing TURBT were allocated to the hospital-based intensive SCI, including weekly meetings, patient education, motivational support, and complimentary nicotine replacement therapy or the standard treatment with very brief advice and referral to the same intensive SCI at a municipality clinic. The primary outcome was smoking cessation at the end of the intervention, assessed through participant interviews. The secondary outcomes included continuous abstinence at three and six months, quality of life, and frailty. Analyses were performed as intention-to-treat. **Results:** After six weeks, 37% in the hospital-based group and 6% in the standard group achieved smoking cessation (*p* = 0.042). At three and six months the quit rates were, 37% and 26% in the hospital-based group, compared to 0% and 0% in the standard group, respectively. No significant differences in quality of life or frailty were found. **Conclusions:** This trial found that hospital-based intensive SCI increased cessation rates compared to standard treatment. It would be valuable to evaluate the impact on postoperative complications, cancer prognosis, and long-term cessation in a sizeable new study.

## Simple Summary

Smoking is a major risk factor for bladder cancer, yet many patients continue to smoke after diagnosis. This study investigated whether a hospital-based smoking cessation program could help patients who are undergoing surgery for bladder tumors to quit smoking. A total of 38 patients participated in this study, with half receiving a 6-week intensive program with weekly meetings, education, motivational support, and free nicotine replacement therapy, while the other half received the standard approach with brief advice and a referral to a similar program at a municipality clinic. The hospital-based program led to significantly higher quit rates, with 37% achieving cessation compared to 6% in the standard treatment group. These findings suggest that intensive hospital-based support can greatly improve smoking cessation rates and may have further benefits for recovery and long-term health. Future research could explore how this approach affects surgical outcomes and long-term cancer prognosis.

## 1. Introduction

Bladder cancer is the ninth most prevalent cancer globally, with an estimated annual incidence of approximately 430,000 cases [1]. At initial diagnosis, approximately 75% of cases are classified as non-muscle invasive bladder cancer (NMIBC), and more than 50% of patients experience recurrence after treatment [2]. The management and surveillance of bladder cancer often involve repeated treatments in the healthcare system, including cystoscopies, transurethral resection of the bladder tumor (TURBT), and intravesical therapies [3], which makes it one of the most costly types of cancer [4].

Smoking is a well-established risk factor for bladder cancer, with approximately 50% of cases attributable to smoking [5,6]. Smoking cessation is strongly recommended in the European Association of Urology treatment guidelines [7]; however, many patients continue to smoke after the diagnosis of NMIBC [8]. The time of diagnosis and surgery may represent a window of opportunity for engaging patients in prehabilitation or lifestyle changes, including smoking cessation [9] thereby, it would be possible to both reduce the progression and relapse as well as postoperative complications. However, few trials investigating prospective smoking cessation interventions (SCIs) within the setting of TURBT have been reported [10]. Intensive SCIs delivered within the surgical setting have demonstrated a cessation rate of about 50% at the time of surgery [11].

To obtain the highest possible quit rate, it is recommended to use intensive SCI for surgical patients. The program should fulfill the American definition [12] as described in the clinical practice guidelines treating tobacco use and dependence which includes education of the smoker, motivational or behavioral counseling, and pharmacotherapy, and be followed through in at least four sessions of at least 10 min each [12,13]. This is also recommended by Health services in other countries including the UK [14] and Sweden [15]. A major difference is that this intensive program is the standard in Denmark, while it in other countries is recommended for specific sub-groups exclusively.

In Denmark, the standardized intensive SCI known as the ’Gold Standard Program’ (GSP) is offered free of charge to all smokers through municipality clinics [16]. This program runs for six weeks and includes weekly meetings that combine motivational [17] and pharmacological support with patient education. The hospital routine includes very brief advice (VBA) [18] delivered as Ask, Advise, Refer to the municipality clinics [19]. VBA is provided to all smokers irrespective of their motivation to quit.

This study aimed to compare the efficacy of intensive SCI delivered at the surgical department with the standard treatment consisting of VBA and referral to the municipality clinic among smokers in relation to TURBT treatment. The secondary outcomes included smoking reduction, frailty, and quality of life.

## 2. Materials and Methods

### 2.1. Study Design

This two-site, parallel-arm, superiority randomized controlled trial was approved by the Danish Scientific Ethical Committee (H-20081571) and the Danish Data Protection Agency (P-2020-95). Informed consent was obtained from all participants, and approval was obtained from the department managers. The trial was registered at ClinicalTrials.gov (NCT04088968) and reported following the CONSORT guidelines [20].

### 2.2. Patients and Recruitment

Consecutive surgical patients at the urology departments at two university hospitals were screened for current smoking habits. Eligible patients received oral and written information about this study. The inclusion criteria were age ≥ 18 years; scheduled for TURBT for diagnostics, resection, and/or disease control; and smoking daily (≥1 cigarette/day). The exclusion criteria included pregnancy; breastfeeding; allergies or contraindications to pharmaceutical support; smoking abstinence exceeding seven days prior to inclusion; cognitive impairments or language challenges prohibiting informed consent; and withdrawal of consent.

The number of participants was 2 × 19 based on the cessation rate of 50% in the hospital-based SCI group versus 10% in the standard group from the literature [21,22] and an 80% power and a 5% significance level.

### 2.3. Randomization

The participants were randomized and allocated at a 1:1 ratio to either the hospital-based SCI or the standard treatment using computer-generated block randomization in block sizes of two or four in a secure REDCap database [23]. The allocation sequence was generated by a person not otherwise involved in this study. Allocation was concealed until enrolment.

This study nurses enrolled eligible participants, administered the hospital-based SCI, and collected the data. Demographic and baseline data were collected before initiating the allocated intervention. Due to the nature of the intervention, it was not feasible to implement blinding for either the participants or the intervention staff. The biomarker and statistical analyses were blinded to group allocation. The participants were allocated to an intensive, individually tailored SCI at the hospital [9] or to the standard treatment.

### 2.4. Interventions

All participants were informed about the link between smoking and bladder cancer, including the benefits of smoking cessation in relation to their disease. They also received information about smoking and smoking cessation in the surgical pathway as well as generally. The smoking status was followed up at six weeks, three, and six months.

#### 2.4.1. Intervention Arm

In the intervention group, all participants were offered a smoking cessation intervention based on the GSP, with a special focus on surgery and bladder cancer, delivered at the hospital. The six-week GSP consisted of weekly counseling, patient education, motivational support, and nicotine replacement therapy (NRT) [24] (Table 1). This manual-based intervention was delivered by trained therapists (the first author LNL and another study nurse) [16].

#### 2.4.2. Standard Treatment

The standard treatment group received VBA [19] and referral to the national Quitline (Stoplinien) which supported them in contacting the municipality clinic for the GSP.

### 2.5. Assessment of Outcomes

The primary outcome was smoking cessation at the end of the six-week intervention, reported by interviews with the participants and validated through urine cotinine levels and carbon monoxide (CO). The secondary outcomes included continuous cessation from the end of the intervention until the three- and six-month follow-ups, point prevalence cessation at three and six months, number of cigarettes, quality of life (EQ-5D) [25], and frailty (modified Fried Frailty Scale) [26] at the end of intervention, three and six months.

Smoking cessation was validated using urinary cotinine, a nicotine metabolite [27], measured with a Waters Acquity UPLC with Xevo TQ-S tandem mass spectrometry with a lower limit of quantitation at 2.0 ng/mL. A cut-off value of 50 ng/mL was applied for discrimination [28]. CO was measured in exhaled breath in parts per million (ppm) [28], with a CO Check Pro from MD Diagnostics, Chatham, Kent, UK or BMC-2000 from SENCO, Ansan-si, Gyeonggi-do, Republic of Korea. We considered measurements of zero to six ppm as validation of self-reported cessation. In accordance with the Russell standard, we assumed that the participants who lost to follow-up were smokers [29].

At baseline, the number of cigarettes was categorized into four groups according to the Fagerström nicotine dependency test [30]: none, 1–10; 11–20; 21–30; and 30+, while the precise number of cigarettes was reported at the follow-up visits. The reduction in consumption was analyzed from baseline to the end of the intervention and at three- and six-month follow-up visits.

Health-related quality of life was measured using the EQ-5D [25]. The EQ-5D includes an overall health rating on a VAS from zero (worst imaginable health) to 100 (best imaginable health) and a summary score based on five dimensions: mobility, self-care, usual activities, pain/discomfort, and anxiety/depression. Each dimension has five levels of severity ranging from none to extreme. We used Danish index values for the summary score [31].

Frailty was assessed using a modified Fried Frailty Scale with five criteria: slowness (timed up and go), weakness (handgrip), exhaustion, weight loss, and low activity [26]. Based on the responses and measurements, a score ranging from zero to five was calculated, and the participants were categorized as non-frail (0), pre-frail (1–2), or frail (3–5) based on a 0–5 scoring system.

Compliance with the GSP was defined as attending ≥ 75% of the sessions.

### 2.6. Statistical Methods

For continuous variables, the medians and ranges were reported, whereas numbers and proportions were reported for categorical variables. The two groups of standard treatment and the hospital-based GSP were compared using Fisher’s exact test for categorical outcomes, and the Mann–Whitney test for continuous outcomes. All analyses were conducted blinded and with intention to treat. A significance level of 5% was used. The data were analyzed using R statistical software version 4.3.0 (2023-04-21).

## 3. Results

### 3.1. Participant Flow

**Figure 1:**
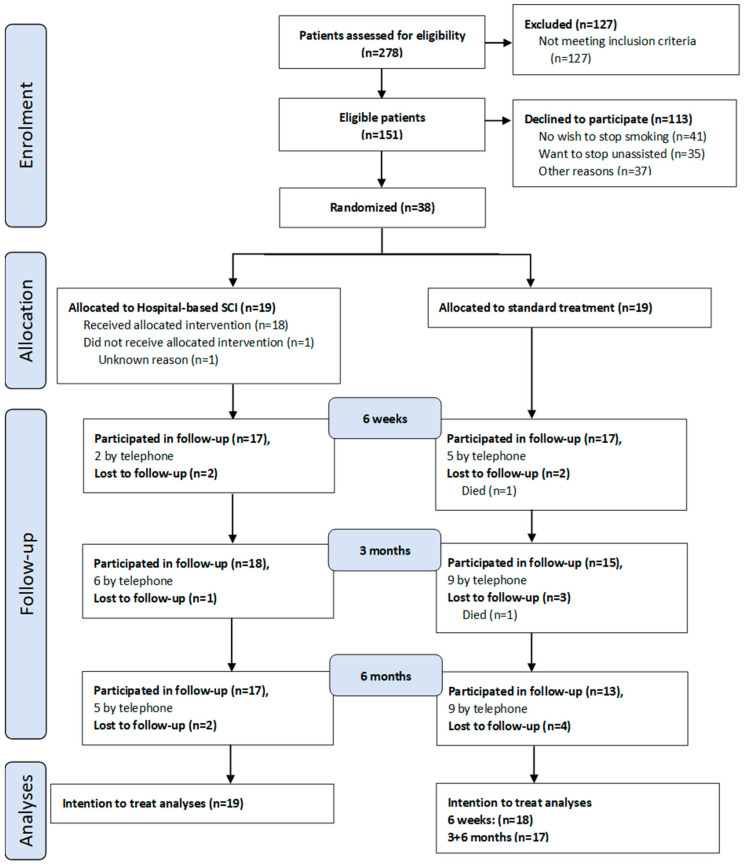
Patient trial profile. SCI = smoking cessation intervention.

All patients undergoing TURBT from December 2021 to March 2024 at Rigshospitalet, Copenhagen University Hospital, were screened for eligibility. To accelerate the recruitment, Herlev Hospital, Copenhagen University Hospital participated from October 2023 until March 2024. Of the 278 patients contacted and screened for eligibility, 127 did not meet the inclusion criteria. Of the remaining patients, 113 declined to participate. Approximately one-third expressed that they did not wish to quit, while another third expressed that they preferred to quit without assistance. Ultimately, 38 patients were included in this study and were randomly assigned into two groups (Figure 1). In the standard treatment group, 1 participant passed away before the six-week follow-up, and another passed away between the six-week and the three-month follow-up; both were censored afterward. Thus, the analyses included 19 participants in the hospital-based arm but 18 in the standard arm at the six-week and 17 at the three- and six-month follow-ups.

The compliance in the hospital-based group was 68%, with 13 attending all six sessions. Only 1 participant attended four sessions, 4 attended one or two sessions, and 1 did not attend any sessions. In contrast, in the standard treatment group, only 4 of 18 participants had contacted or engaged with the municipality clinic at the six-week follow-up.

As presented in Figure 1, some participants did not complete the follow-ups. Furthermore, several were not able to participate in person leading to missing data on variables requiring measurement and urine samples. The baseline characteristics are described in Table 2.

### 3.2. Primary Outcome: Smoking Cessation at the Six-Week Follow-Up

At the end of the intervention, 7/19 (37%) participants in the hospital-based group and 1/18 (6%) in the standard group reported successful quitting (*p* = 0.04) (Table 3). Validated by CO measurement, the numbers were 5/19 (26%) and 1/18 (6%), respectively (*p* = 0.18). Due to the current NRT, the validation with urine cotinine was positive for all; however, one successful quitter in the standard group missed the urine sampling.

### 3.3. Secondary Outcomes

At three and six months, 37% and 26% of the participants, respectively, in the hospital-based group and none in the standard group were continuously smoke-free. The point prevalence cessation was 47% and 32%, respectively, in the hospital-based group, and 0% and 6%, respectively, in the standard group (Table 3). The cessation rates validated by urine cotinine and CO were lower.

There were no significant differences regarding quality of life (EQ-5D) or frailty scores between the groups at the follow-up visits (Table 3).

**Figure 2:**
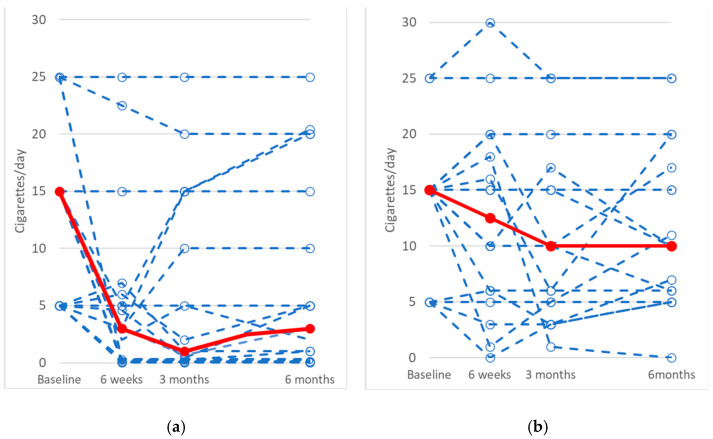
Trends in cigarette use at baseline and follow-up time points. Each dashed blue line represents an individual participant’s cigarette consumption, while the solid red line represents the median cigarette use at each time point. (**a**): Hospital-based group; (**b**): Standard treatment group. Baseline cigarette use was categorized as: 1–10/day = 5, 11–20/day = 15, and 21–30/day = 25. Missing follow-up values were imputed using the participant’s baseline value.

Cigarette use at baseline and follow-up visits is presented as spaghetti plots in Figure 2a,b for the hospital-based group and the standard treatment group, respectively. Figure 2a demonstrates a reduction in median cigarette use over time in the intervention group, which contrasts with the more modest changes observed in the control group shown in Figure 2b.

### 3.4. Harms

Nine participants experienced mild side effects from NRT, including skin irritation (*n* = 4); hiccups, nausea, or upset stomach (*n* = 4); dizziness (*n* = 1); and sleep disturbances (*n* = 1). These side effects did not cause discontinuation of use; however, the type of NRT was often changed. The use of NRT and its potential side effects were discussed at each interventional meeting, allowing for treatment to be tailored and adjusted to individual patient needs. Side effects were managed through education on proper product use or by switching to an alternative NRT product.

## 4. Discussion

The smoking cessation rate was significantly higher after the hospital-based GSP than in the standard treatment group at the end of the intervention and at longer follow-up. Correspondingly, the number of cigarettes smoked was significantly reduced. Whereas the other outcomes were not different between the groups.

Abstinence rates at the end of the intervention were slightly lower than those reported in previous intensive SCI studies within surgical populations [11]. The low degree of cessation observed in the standard treatment group may be due to only a few participants attending the intensive SCI at the municipality clinics. Consequently, the standard treatment group outcomes are similar to those typically achieved using the VBA approach alone in clinical settings [18].

Within the field of urology, few studies have investigated the efficacy of SCI for patients treated for bladder cancer [10]. A randomized controlled study using the same type of intervention has reported higher cessation rates among 104 patients undergoing radical cystectomy and receiving intensive smoking and/or alcohol intervention. At the end of the intervention, 51% in the intervention group were continuously abstinent compared to 27% in the usual treatment group [32]. In contrast, a prospective trial involving 179 outpatients with various urological conditions compared a brief SCI of less than five minutes to usual care and reported only 12% and 3% cessation, respectively, at the one-year follow-up [33]. We did not identify any studies reporting on intensive SCI in relation to TURBT within a randomized controlled trial design.

In non-urology settings, the GSP was evaluated in RCTs involving patients undergoing hip and knee replacement and general surgery. The reported quit rates at the end of the intervention in the GSP groups were 60% and 40%, respectively [34,35]. In comparison, our study found lower smoking cessation rates in the hospital-based SCI. This suggests that the smoking cessation intervention outcomes may differ depending on the patient population and the clinical context.

It is important to offer the most effective program, as cessation periods exceeding 10 years have been associated with a reduced risk of recurrence [36]. Additionally, evidence suggests that smoking cessation may improve recurrence-free survival in patients with NMIBC [37].

Despite using the same GSP for SCI in the municipality clinics and in the hospital-based intervention, only four participants from the standard group approached the municipality clinic within the 6-week follow-up. The low adherence rates to the smoking cessation intervention in the standard treatment group may have been influenced by several factors, including lack of motivation, logistical barriers, and disappointment with being assigned to the control group. Previous research suggests that being allocated to the control group in a randomized controlled smoking cessation trial may have resulted in feelings of disappointment [38], which may explain not only the low interest in joining the SCI in the municipality but also the greater number of participants not attending follow-up in person. To improve adherence in future studies, several strategies could be explored including, motivational phone calls, SMS reminders, and more active guidance of patients in the municipality clinics.

In the hospital-based group, most of the participants had participated in at least one GSP meeting and more than two-thirds had participated in all the meetings. A hospital-based intervention delivered by a clinician in concordance with treatment may lead to feelings of the SCI as an integral component of the treatment pathway, benefitting adherence [39,40].

In Denmark, VBA followed by SCI in the municipality are the recommended approaches for addressing smoking among hospital patients. Despite its endorsement, widespread adoption remains challenging, and only a limited part of smokers are referred and even fewer enter the municipality clinics [19]. In this study, we found that it is feasible to deliver the SCI at the hospital, as the participants who enrolled were willing to attend the counseling sessions, even when these were not arranged in relation to other hospital appointments. However, it is important to note that a substantial number of patients declined to participate, which may indicate potential barriers to engagement that warrant further investigation.

Behavioral change is often a dynamic process with individuals progressing through different stages of readiness for change [41]. As mentioned above, receiving a severe diagnosis, and undergoing surgery may create a window of opportunity for behavior change [9].

The practical application of the results as part of the surgical intervention would lead to perioperative quitting of about one-third of the smokers still smoking when referred to the hospital. In addition, the majority of those have successfully quit at longer time as well and thereby get the benefits in both the short and longer term. It would, however, require preoperative attention from the clinicians regarding smoking in line with co-morbidity, priority of implementation of a new guideline, smoke-free culture, and policy. The results also indicate that the trained therapist delivering the intensive SCI to surgical patients should stay at the hospital and integrate the sessions into the already planned contacts—as far as possible. Thereby, the SCI would follow the patients as in the intervention group instead of the opposite.

In our study, many patients continued to smoke, and they should be offered information and intensive SCI, repeatedly throughout the TURBT treatment pathway to enhance engagement and improve smoking cessation outcomes.

A strength of our study is the randomized controlled design and the manual-based SCI [42]. We planned to strengthen this study by validating successful cessation by CO measurement and urine cotinine [29]. However, the use of NRT interfered with the usual cut-off values for urine cotinine [28]. The measurement of CO is useful and a widely accepted method for validating smoking cessation, including in participants using NRT. CO has a short half-life and reflects recent smoking [28,29]. Alternatively, a biomarker specific to tobacco, such as the alkaloids anabasine or anatabine, may be valuable to consider in future studies [28,43].

Another bias is the participants not being followed up in person, with more participants in the standard group having follow-ups conducted by telephone.

Furthermore, the standard treatment may have exceeded the typical VBA and referral definitions as this group received careful patient information in addition. Nevertheless, this seems not to have impacted the standard group. This study also has several limitations since 75% of the eligible patients did not want to be included. About 25% of the eligible patients stated that they intended to quit without participating in a smoking cessation intervention and another 25% stated that they had no desire to quit smoking. To improve recruitment rates in future studies potential strategies could include enhancing patient education on the benefits of participating in a structured cessation program. Despite enrolment challenges being general in the SCI trials [11], the generalization of the results should be considered thoroughly as they are connected to a university hospital in a Scandinavian high-income country and with treatment free of charge.

### Perspectives

This study demonstrated the feasibility of hospital-based intensive SCI, suggesting that it may enhance patient engagement and adherence. These findings indicate that integration into the clinical care pathway may enhance patient engagement.

Integrating hospital-based CSIs into routine practice requires consideration of cost, resource allocation, and scalability in the short and long term. In the short term, successful implementation in the surgical setting would require a reallocation of resources, both human and economic, while the short-term benefit includes a reduction in postoperative complications. In the longer term, the resource consumption will lower due to reduction of recurrence and aggravation of the cancer disease as well as for other smoking-related diseases and conditions.

Smoking cessation has a tremendous effect on clinical and public health. Scalability to all surgical settings would lead to a reduction of postoperative complications [21] in the short term for the benefit of individual patients and healthcare. The reduced smoking rate would further impact the public health in the society at large.

An effective smoking cessation intervention with high patient engagement and adherence may increase the number of patients who successfully quit smoking among those who undergo TURBT, potentially easing the burden of disease. From a clinical and management perspective, adhering to international guidelines is essential.

Most studies on SCI in surgical settings have been conducted in high-income countries such as the US, Canada, Australia, and Western Europe [11,44]. This highlights the need for research in low and middle-income countries, where healthcare infrastructure, resources, and smoking cessation support may differ greatly.

As this seems to be the first study within this area future research should focus on optimizing the delivery of intensive SCIs to surgical patients and exploring strategies to improve recruitment. Conducting a sizeable RCT to evaluate the impact of intensive SCI on postoperative complications, cancer prognosis, and long-term cessation beyond the six-month follow-up would provide valuable insights into both clinical outcomes and sustained smoking cessation. Further cohort studies on recurrence rates and progression would also add to the clinical applicability.

Since our study found lower cessation rates with the hospital-based GSP than expected, we suggest that future research use a sample size estimation based on our finding; 37% in the hospital-based SCI group versus an expected 6% in the treatment-as-usual group. A future study would then require 2 × 24 participants while more conservative estimates of 30% versus 10% would require 2 × 59 patients to achieve statistical power.

## 5. Conclusions

This trial compared hospital-based intensive SCI with standard treatment for patients undergoing TURBT. Significantly more patients in the hospital-based group reported cessation at the end of the intervention, and after three months, but not at longer follow-up. This study highlights the feasibility and potential benefits of hospital-based SCIs.

## Figures and Tables

**Table 1:** The six-week GSP for smoking cessation in the surgical setting with weekly counseling meetings.

Patient Education Program
First meeting:
Brief information on the harmful health effects of smoking, the risk of developing and progression of bladder cancer as well as postoperative complications
Level of motivation, ambivalence, pros, and cons for cessation and continued smoking
Second meeting:
Addiction including withdrawal symptoms during cessation
Nicotine replacement therapy (NRT) individually targeted
Third meeting:
Risk of relapse and management of high-risk situations
Fourth meeting:
Maintaining continuous smoking cessation
Fifth meeting:
Handling high-risk situations for relapse in the future and NRT tapering
Sixth meeting:
Handling high-risk situations for relapse in the future and NRT tapering
At each meeting:
Follow-up on smoking status, successes, and challenges during the last week
NRT tailored to patient preferences and levels of nicotine addiction
Validation by CO and urine cotinine

**Table 2:** Baseline characteristics of participants. Presented as the number (%) or median [range].

	Intervention Arm, *n* = 19	Standard Arm, *n* = 19	Total *n* = 38
**Patient characteristics**			
Age, years	63 [44–80]	70 [39–77]	63 [39–80]
Male	15 (79)	14 (74)	29 (76)
Living alone	12 (63)	10 (53)	22 (58)
Not married or cohabiting	12 (63)	9 (47)	21 (55)
Education: Short or none ^a^	3 (16)	4 (21)	7 (18)
Quality of life:			
EQ-5D summary score ^b^	0.91 [0.25–1]	0.90 [0.19–1]	0.90 [0.19–1.00]
EQ-5D VAS ^c^	80 [25–100]	75 [20–100]	75 [20–100]
Frailty level			
Frail	1 (5)	4 (21)	5 (13)
Prefrail	9 (47)	8 (42)	17 (45)
Non-frail	9 (47)	6 (32)	15 (39)
Missing data	-	1(5)	1 (3)
**Smoking history and status**			
Pack years ^d^	40 [12–90]	56 [16–78]	41 [12–90]
Fagerströms score ^e^	4 [0–8] ^1^	5 [2–8]	4 [0–8]
Cigarettes per day:			
1–10	8 (42)	4 (21)	12 (32)
11–20	6 (32)	13 (68)	19 (50)
21–30	4 (21)	2 (11)	6 (16)
Missing data	1 (5)	-	1 (3)
Previous quit attempts (last 10 years)			
No	7 (37)	8 (42)	15 (40)
Yes	12 (63)	11 (58)	23 (60)
Encouraged to stop smoking during the last year ^2^:			
Primary healthcare	12 (63)	13 (68)	25 (66)
Secondary healthcare	14 (73)	13 (68)	27 (71)
None of the above	5 (26)	3 (16)	8 (21)
**Lifestyle factors**			
BMI	27.7 [17.5–35.1]	25.1 [18.7–39.1]	26.1 [17.5–39.1]
BMI ≥ 30	5 (26)	3 (16)	8 (21)
Malnutrition ^f^	2 (11)	1 (5)	3 (8)
Alcohol consumption > 14 AU/week	1 (5)	4 (21)	5 (13)
Physical activity < 30 min/day	5 (26)	6 (32)	11 (29)
**Disease characteristics:**			
ASA class by anaesthesiologist.			
1–2	13 (68)	11 (58)	24 (63)
3	6 (32)	8 (42)	14 (37)
CCI age-adjusted	3 [0–7]	4 [0–9]	4 [0–9]
Pathology TURBT			
NMIBC	13 (68)	16 (84)	29 (76)
MIBC	4 (21)	0	4 (11)
Benign	2 (11)	2 (11)	4 (11)
Non urothelial cancer	0	1 (5)	1 (3)

**Abbreviations:** ASA = American society of anesthesiologists. AU = Alcohol units. BMI = Body mass index. CCI = Charlson comorbidity index. NMIBC = Non-muscle invasive bladder cancer. MIBC = Muscle invasive bladder cancer. Notes: ^a^ Short or no education level = no education except primary school or short work-related courses. ^b^ EQ-5D summary score = A summary score based on five dimensions ranging from − 0.757 to 1, with 1 representing full health. ^c^ EQ-5D VAS = Overall health rating on a visual analog scale from 0 (worst imaginable health) to 100 (best imaginable health).^d^ Pack years = (Number of cigarettes smoked per day) × (Number of years smoked)/20. ^e^ Fagerströms score = Test for Nicotine Dependence. The score ranges from 0 to 10, with higher scores indicating a stronger dependence on nicotine. ^f^ Malnutrition = Weight loss >5% in 1 month or Food intake 0–25% of need in the past week or BMI <18.5 and impaired condition. ^1^ One participant had missing data of quantity per day. ^2^ Multiple answers possible.

**Table 3:** Outcomes after hospital-based smoking cessation intervention (IG) vs. Standard treatment (CG). Presented as the number (%) or median [range].

Outcome	6 Weeks			3 Months			6 Months		
	IG *n* = 19	CG *n* = 18	*p*-Value	IG *n* = 19	CG *n* = 17	*p*-Value	IG *n* = 19	CG *n* = 17	*p*-Value
Continuous cessation	-	-	-	7 (36.8)	0 (0)	0.01	5 (26.3)	0 (0)	0.05
Point prevalence cessation ^a^	7 (36.8)	1 (5.5)	0.04	9 (47.4)	0 (0)	<0.00	6 (31.6)	1 (5.8)	0.09
Cigarettes per day:			0.02			0.02			0.37
0	7 (36.8)	1 (5.6)		9 (47.4)	0 (0)		6 (31.6)	1 (5.8)	
1–10	7 (36.8)	7 (38.9)		4 (21.1)	9 (52.9)		4 (21.1)	7 (41.1)	
11–20	0 (0)	6 (33.3)		4 (21.1)	4 (23.5)		4 (21.1)	4 (23.5)	
21–30	1 (5.3)	2 (11.1)		1 (5.3)	2 (11.7)		1 (5.3)	1 (5.8)	
>30	0 (0)	0 (0)		0 (0)	0 (0)		0 (0)	0 (0)	
Missing	4 (21.1)	2 (11.1)		1 (5.3)	2 (11.7)		4 (21.1)	4 (23.5)	
Quality of life:									
EQ-5D summary score ^b^	0.95 [0.25–1.00]	0.92 [0.46–1.00]	0.89	0.90 [0.35–1.00]	0.88 [0.25–1.00]	0.92	0.88 [0.13–1.00]	0.91 [0.37–1.00]	0.87
EQ-5D VAS ^c^	85 [35–95]	80 [50–100]	0.56	80 [40–98]	80 [40–100]	0.25	90 [45–100]	83 [50–100]	0.96
Missing	4	2		4	2		2	6	
Frailty score ^d^	1 [0–3]	1 [0–3]	0.39	1 [0–3]	0 [0–3]	0.33	1 [0–2]	0 [0–3]	0.96
Missing	4	7		7	11		7	12	

**Abbreviations:** IG = Intervention group (hospital-based group), CG = control group (standard treatment group). Notes: ^a^ Point prevalence = No smoking for the past seven days. ^b^ EQ-5D summary score = A summary score based on five dimensions ranging from − 0.757 to 1, with 1 representing full health. ^c^ EQ-5D VAS = Overall health rating on a visual analog scale from 0 (worst imaginable health) to 100 (best imaginable health [40–100). ^d^ Frailty score = score of 0–5, 0 indication non-frail, 1–2 pre-frail, and 3–5 frail.

## Data Availability

The data that support the findings of this study are available from the corresponding author, L.N.L., upon reasonable request.

## Abbreviations

The following abbreviations are used in this manuscript:

SCI	Smoking cessation intervention.
TURBT	Transurethral resection of the bladder tumor.
NMIBC	Non-muscle invasive bladder cancer.
GSP	Gold standard program.
VBA	Very brief advice.
NRT	Nicotine replacement therapy.
CO	Carbon monoxide.
PPM	Parts per million.
ASA	American society of anesthesiologists.
AU	Alcohol units.
BMI	Body mass index.
CCI	Charlson comorbidity index.
MIBC	Muscle invasive bladder cancer.
VAS	Visual analog scale.
IG	Intervention group.
CG	Control group.
